# Supplementary material for: A robust and tunable Luttinger liquid in correlated edge of transition-metal second-order topological insulator Ta2Pd3Te5
Source: Nat Commun. 2023 Nov 23;14:7647. doi: 10.1038/s41467-023-43361-5 (PMC10667360; doi:10.1038/s41467-023-43361-5)
Supplement: Supplementary file 1 — Supplementary information [file 41467_2023_43361_MOESM1_ESM.pdf]

Supplementary Information for

A robust and tunable Luttinger liquid in correlated edge of transition-metal second-order topological insulator $Ta_2Pd_3Te_5$

Anqi Wang[1,2,†], Yupeng Li[1,†], Guang Yang[1,†], Dayu Yan[1,†], Yuan Huang[3], Zhaopeng Guo[1], Jiacheng Gao[1,2], Jierui Huang[1,2], Qiaochu Zeng[1], Degui Qian[1], Hao Wang[1], Xingchen Guo[1,2], Fanqi Meng[1], Qinghua Zhang[1,4], Lin Gu[1,2,5], Xingjiang Zhou[1,2,5], Guangtong Liu[1,5], Fanming Qu[1,5], Tian Qian[1,5], Youguo Shi[1,2,5*], Zhijun Wang[1,2*], Li Lu[1,2,5*], Jie Shen[1,5*]

[1]Beijing National Laboratory for Condensed Matter Physics, Institute of Physics, Chinese Academy of Sciences, Beijing 100190, China

[2]School of Physical Sciences, University of Chinese Academy of Sciences, Beijing 100049, China

[3]Advanced Research Institute of Multidisciplinary Science, Beijing Institute of Technology, Beijing 100081, China

[4]Yangtze River Delta Physics Research Center Co. Ltd, Liyang 213300, China

[5]Songshan Lake Materials Laboratory, Dongguan 523808, China

†These authors contributed equally to this work

*Corresponding author. Email: ygshi@iphy.ac.cn (Y.-G.S.), wzj@iphy.ac.cn (Z.-J.W.), lilu@iphy.ac.cn (L.L.), shenjie@iphy.ac.cn (J.S.)

Supplementary Table 1. The irreps table of Gamma point of space group 59.

	GM1+	GM1-	GM2+	GM2-	GM3+	GM3-	GM4+	GM4-
{E|0,0,0}	1	1	1	1	1	1	1	1
{C_{2x}|0,1/2,1/2}	1	1	1	1	-1	-1	-1	-1
{C_{2y}|0,1/2,0}	1	1	-1	-1	1	1	-1	-1
{C_{2z}|0,0, 1/2}	1	1	-1	-1	-1	-1	1	1
{-1|0,0,0}	1	-1	1	-1	1	-1	1	-1
{M_x|0,1/2, 1/2}	1	-1	1	-1	-1	1	-1	1
{M_y|0, 1/2, 0}	1	-1	-1	1	1	-1	-1	1
{M_z|0, 0,1/2}	1	-1	-1	1	-1	1	1	-1

Supplementary Table 2. The irreps table of Y point of space group 59.

	Y1	Y2
{E|0,0,0}	$\begin{pmatrix}1 & 0\\0 & 1\end{pmatrix}$	$\begin{pmatrix}1 & 0\\0 & 1\end{pmatrix}$
{C_{2x}|0,1/2,1/2}	$\begin{pmatrix}0 & 1\\1 & 0\end{pmatrix}$	$\begin{pmatrix}0 & 1\\1 & 0\end{pmatrix}$
{C_{2y}|0,1/2,0}	$\begin{pmatrix}1 & 0\\0 & -1\end{pmatrix}$	$\begin{pmatrix}-1 & 0\\0 & 1\end{pmatrix}$
{C_{2z}|0,0, 1/2}	$\begin{pmatrix}0 & -1\\1 & 0\end{pmatrix}$	$\begin{pmatrix}0 & 1\\-1 & 0\end{pmatrix}$
{-1|0,0,0}	$\begin{pmatrix}1 & 0\\0 & -1\end{pmatrix}$	$\begin{pmatrix}1 & 0\\0 & -1\end{pmatrix}$
{M_x|0,1/2, 1/2}	$\begin{pmatrix}0 & -1\\1 & 0\end{pmatrix}$	$\begin{pmatrix}0 & -1\\1 & 0\end{pmatrix}$
{M_y|0, 1/2, 0}	$\begin{pmatrix}1 & 0\\0 & 1\end{pmatrix}$	$\begin{pmatrix}-1 & 0\\0 & -1\end{pmatrix}$
{M_z|0, 0,1/2}	$\begin{pmatrix}0 & 1\\1 & 0\end{pmatrix}$	$\begin{pmatrix}0 & -1\\-1 & 0\end{pmatrix}$

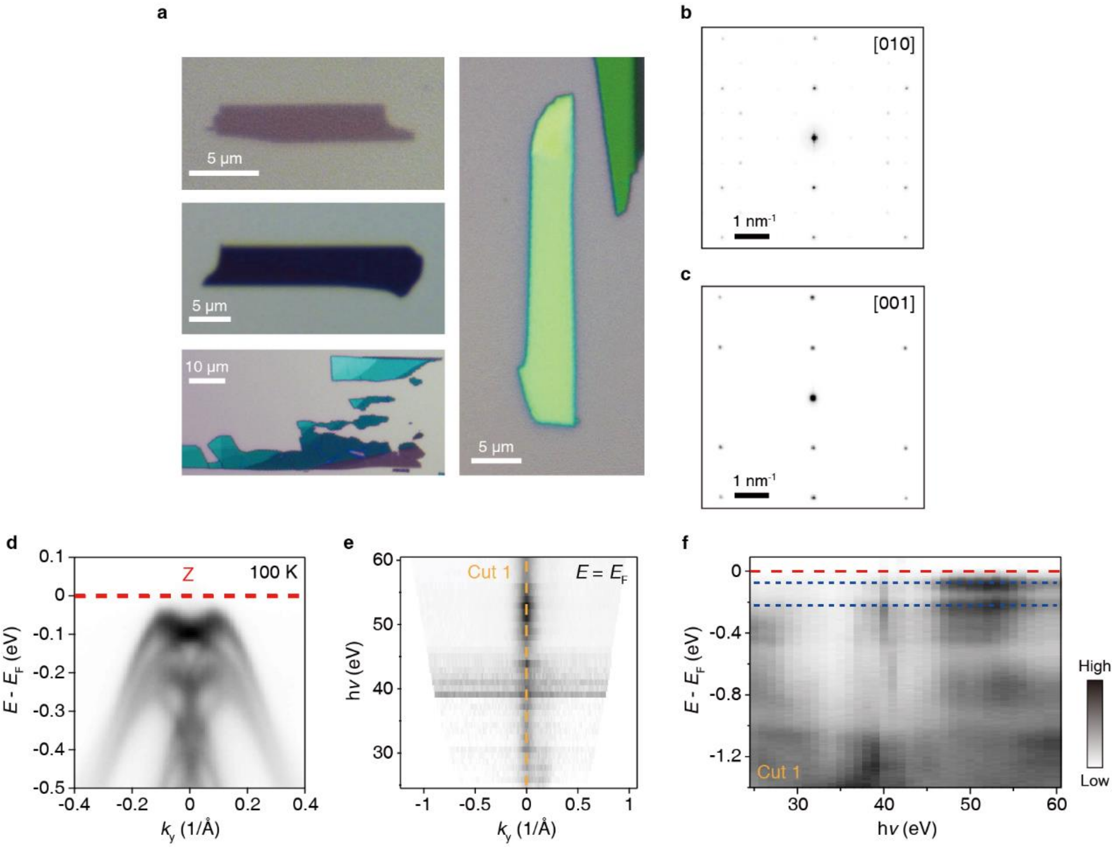

Supplementary Fig. 1 Optical image, STEM image and ARPES data for $Ta_2Pd_3Te_5$. a, Optical images of $Ta_2Pd_3Te_5$ thin films with different thicknesses. The uniform edges indicate the quasi-one-dimensional nature of $Ta_2Pd_3Te_5$. **b,c,** Fast Fourier transformed STEM images of $Ta_2Pd_3Te_5$ single crystal along [010] **(b)** and [001] **(c)** direction which give lattice parameters a ~ 13.9 Å and b ~ 3.7 Å. **d,** ARPES intensity plot of the band structure along the $\bar{Z}$ - $\bar{T}$ direction at T = 100 K. **e,** Intensity plot of ARPES data at $E = E_F$ collected in a range of photon energies from 25 to 60 eV. **f,** Intensity plot of ARPES data along cut 1.

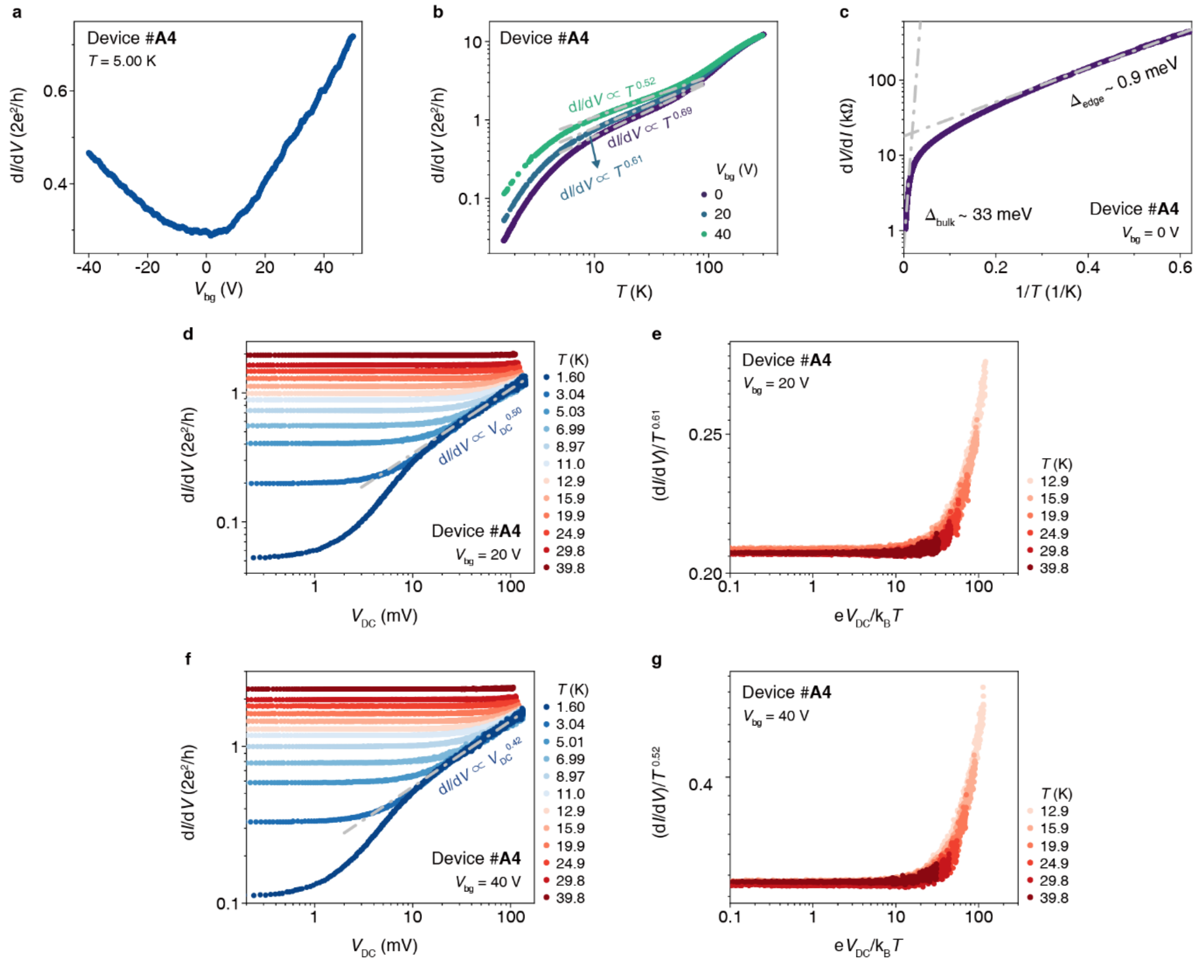

Supplementary Fig. 2 Electrical transport measurement for device #A4. a, dI/dV versus V_{bg} measured at T = 5.00 K. **b,** Log-log plot of temperature dependence dI/dV at different V_{bg}. The gray dot dash lines show the power-law behaviors. **c,** log (dV/dI) v.s. 1/T plot of the temperature dependence resistance at V_{bg} = 0 V. **d,** dI/dV versus V_{DC} measured at differential T with V_{bg} = 20 V. **e,** Curves form T = 12.9 K to 39.8 K in **(d)** are plotted as scaled conductance (dI/dV)/$T^{0.61}$ versus scaled temperature eV_{DC}/k$_{\mathrm{B}}T$. **f,** dI/dV versus V_{DC} measured at differential T with V_{bg} = 40 V. **g,** Curves form T = 12.9 K to 39.8 K in **(f)** are plotted as scaled conductance (dI/dV)/$T^{0.52}$ versus scaled temperature eV_{DC}/k$_{\mathrm{B}}T$.

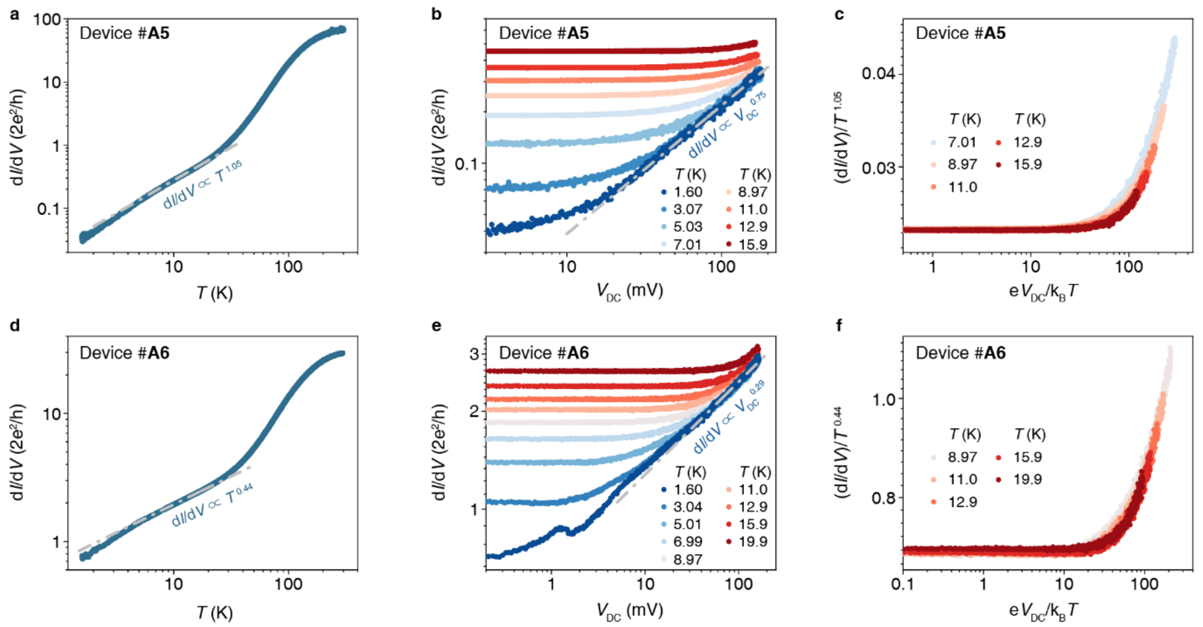

Supplementary Fig. 3 Luttinger liquid behaviors in devices #A5 and #A6. a, Temperature dependence dI/dV of device #A5. **b,** dI/dV versus V_{DC} of device #A5 at differential T. **c,** Curves form T = 7.01 K to 15.9 K in **(b)** are plotted as scaled conductance (dI/dV)/$T^{1.05}$ versus scaled temperature eV_{DC}/k$_B$$T$. **d,** Temperature dependence dI/dV of device #A6. **e,** dI/dV versus V_{DC} of device #A6 at differential T. **f,** Curves form T = 8.97 K to 19.9 K in **(e)** are plotted as scaled conductance (dI/dV)/$T^{0.44}$ versus scaled temperature eV_{DC}/k$_B$$T$.

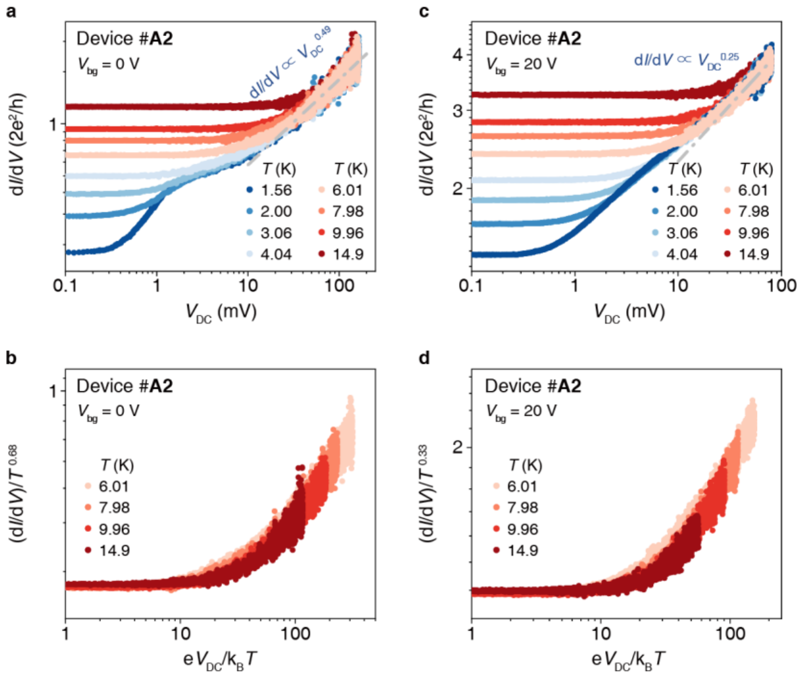

Supplementary Fig. 4 Luttinger liquid behavior in device #A2. a, dI/dV versus V_{DC} at differential T with V_{bg} = 0 V. **b,** Curves form T = 6.01 K to 14.9 K in **(a)** are plotted as scaled conductance (dI/dV)/$T^{0.68}$ versus scaled temperature eV_{DC}/k$_B T$. **c,** dI/dV versus V_{DC} at differential T with V_{bg} = 20 V. **d,** Curves form T = 6.01 K to 14.9 K in **(c)** are plotted as scaled conductance (dI/dV)/$T^{0.33}$ versus scaled temperature eV_{DC}/k$_B T$.

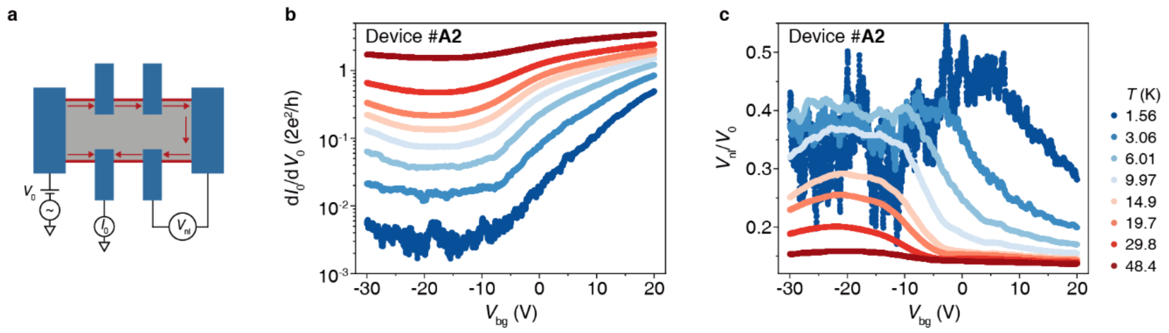

Supplementary Fig. 5 Nonlocal measurement for device #A2. a, Schematic nonlocal measurement configuration with Hall bar shape contacts (blue) on a $Ta_2Pd_3Te_5$ thin film (gray). The red lines are edges of the thin film and red arrows are current flow along edges. **b,** Local differential conductance dI_0/dV_0 measured in device #A2 as a function of V_{bg} at differential T. **c,** Nonlocal voltage ratio V_{nl}/V_0 versus V_{bg} at different T.

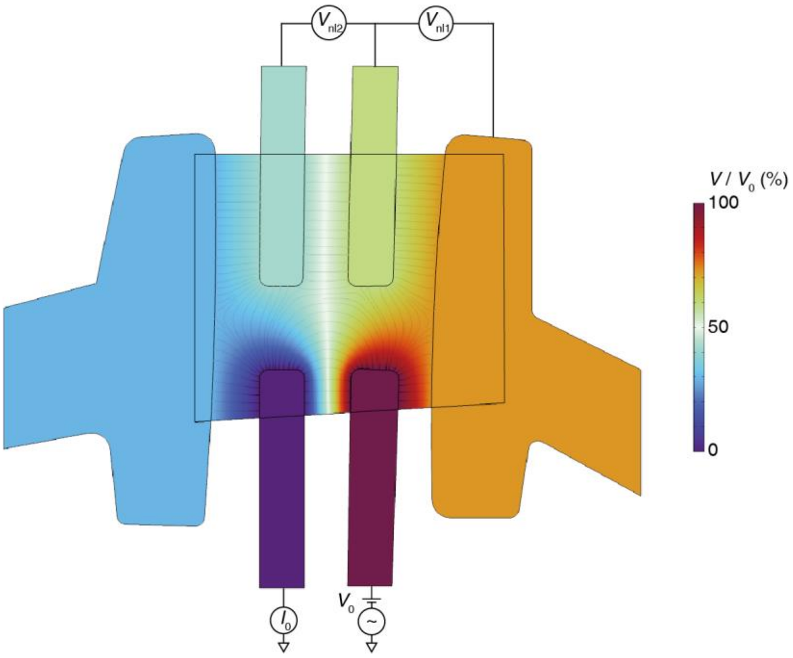

Supplementary Fig. 6 Simulation of potential distribution in homogeneous 2D ohmic resistivity situation for device #A3. It gives V_{nl1}/V_0 ~ 13.2% and V_{nl2}/V_0 ~ 17.3%.

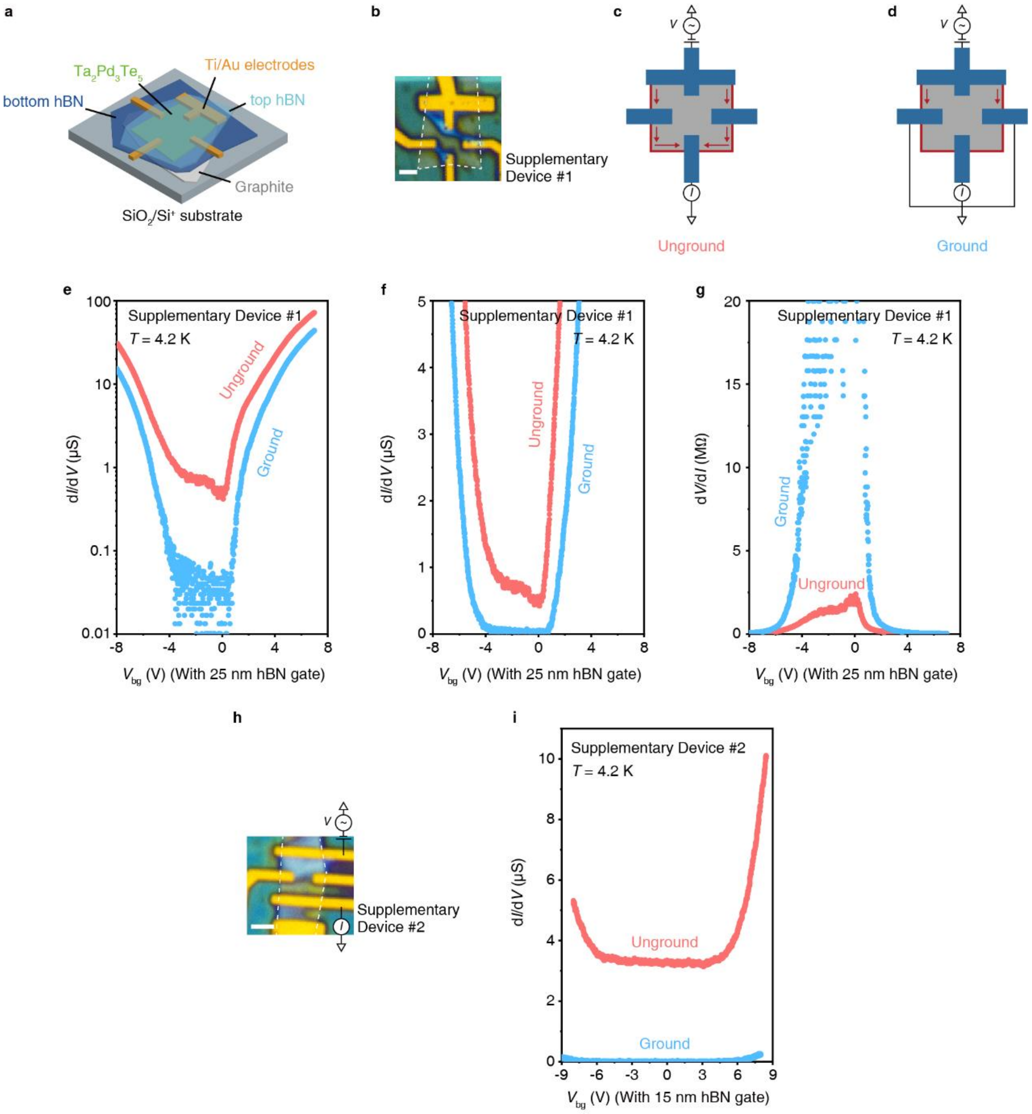

Supplementary Fig. 7 Validation of edge state. a, Sketch map of the hBN bottom gate devices (see 'Devices fabrication' section in Methods for detail). **b,** Optical image of Supplementary device #1 with hBN bottom gate. The dashed white line highlights the $Ta_2Pd_3Te_5$ thin film. The contrast color regions are due to the unevenness of the top hBN induced by the thick (~ 70 nm) Ti/Au electrodes which are insulating and serve only as protecting layers. So, they do not influence the transport behavior. (All other devices except device #A6 are coated with PMMA as protecting layer with clean and smooth surfaces indicating the high quality, e.g. inset in Fig. 2b,c, Fig. 3a, etc.) Scale bar, 2 μm. **c,d,** Schematic measurement configuration of the unground **(c)** and ground **(d)** configuration. The red lines are edges of the thin film and red arrows are current flow along edges. The edges are short out when grounding the side contacts and current can only flow through the bulk. **e,** $\mathrm{d}I/\mathrm{d}V$ versus V_{bg} in unground (red) and ground (blue) configuration, the conductance around CNP is finite in unground configuration, compared with the close-to-zero conductance when grounding the edge. **f,** The same data as **(e)**, but magnify the *y*-axis. **g,** The same data as **(e)**, but plotted as $\mathrm{d}V/\mathrm{d}I$ versus V_{bg}. **h,i,** The same technique as above are used in Supplementary device #2 to validate the existence of edge states.

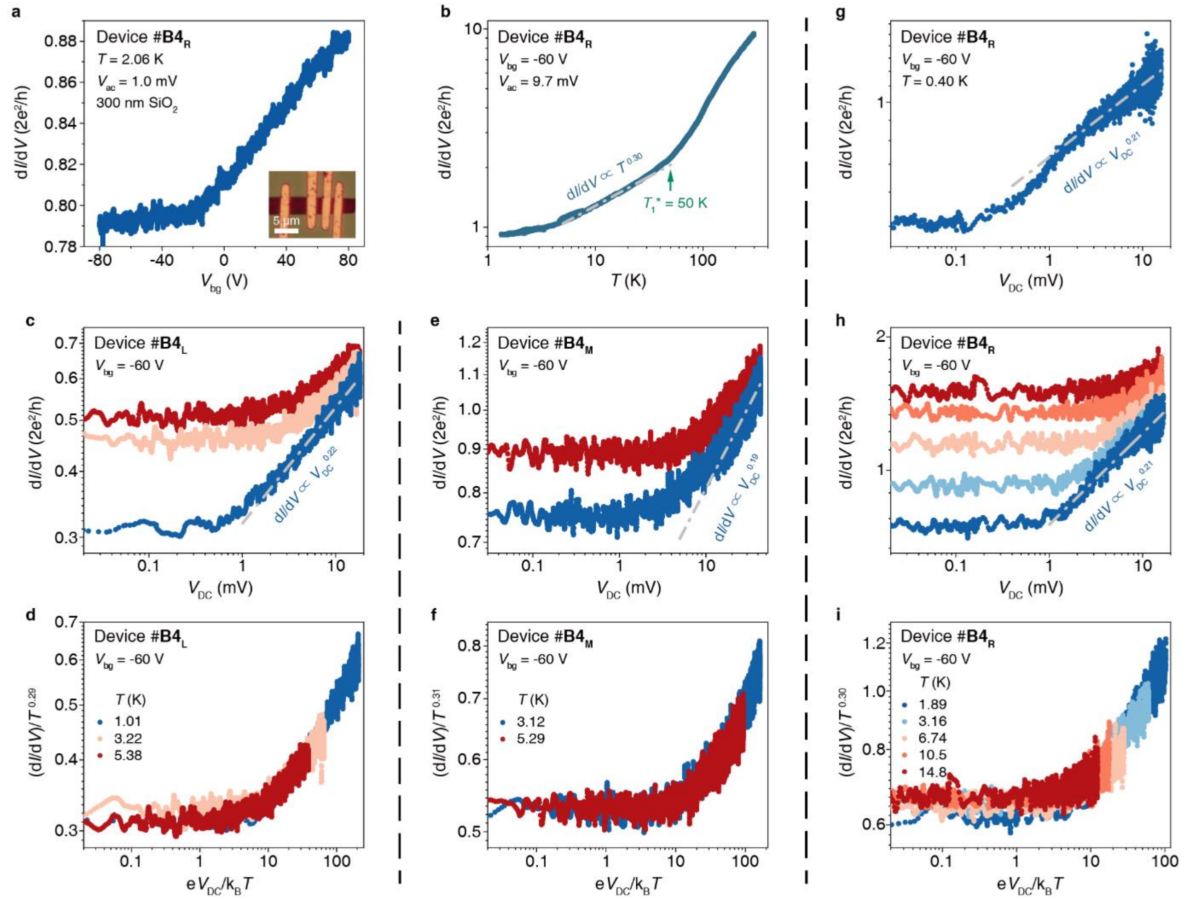

Supplementary Fig. 8 Luttinger liquid behaviors in device #B4. a, dI/dV versus V_{bg} at T = 2.06 K measured with two-terminal configuration using the right two leads in device #B4 with 300 nm SiO_2 bottom gate. Inset: Optical image of device #B4. **b,** dI/dV versus T at V_{bg} = -60 V measured with two-terminal configuration using the right two leads in device #B4. Note that the slight saturation below ~ 6 K is due to large a.c. voltage we applied. **c,d,** dI/dV v.s. V_{DC} **(c)** and (dI/dV)/$T^{0.29}$ v.s. eV_{DC}/k$_{\mathrm{B}}T$ **(d)** measured at V_{bg} = -60 V with two-terminal configuration using the left two leads in device #B4. **e,f,** dI/dV v.s. V_{DC} **(e)** and (dI/dV)/$T^{0.31}$ v.s. eV_{DC}/k$_{\mathrm{B}}T$ **(f)** measured at V_{bg} = -60 V with two-terminal configuration using the two middle leads in device #B4. **g,** dI/dV v.s. V_{DC} measured at V_{bg} = -60 V and T = 0.40 K with two-terminal configuration using the right two leads in device #B4. It exhibits sight edge gap behavior at T = 0.40 K which is not observable above 1.89 K**. h,i,** dI/dV v.s. V_{DC} **(h)** and (dI/dV)/$T^{0.30}$ v.s. eV_{DC}/k$_{\mathrm{B}}T$ **(i)** measured at V_{bg} = -60 V with two-terminal configuration using the right two leads in device #B4. The gray dot dash lines show power-law behaviors. The universal scaling behaviors and consistent power law exponents α indicate that Littinger liquid behavior is the intrinsic property of $Ta_2Pd_3Te_5$.

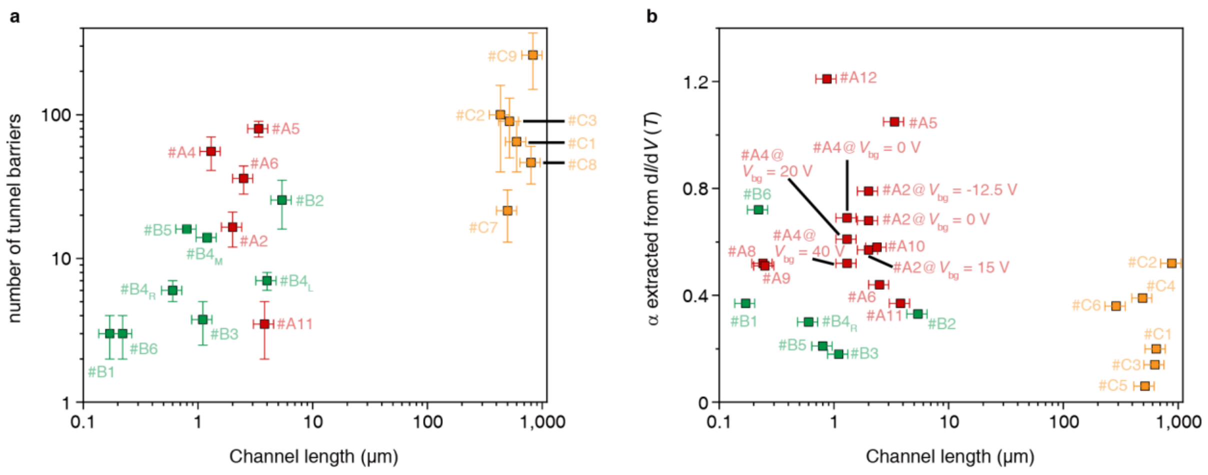

Supplementary Fig. 9 Statistical result of the relation between number of tunnel barriers (a)/power exponents (b) and channel length of the devices/samples. The error bars in *x*-axis reflect the uncertainty from channel length measurement and error bars in *y*-axis in **(a)** reflect the uncertainty of the extracted numbers. Note that the d*I*/d*V* (V_{DC}) curves used to extract the number of tunnel barriers of bulk samples in **(a)** and d*I*/d*V* (*T*) curves used to extract α of bulk samples in **(b)** are measured in different cryostats. The leads spacing are different between two measurements. So, the channel length of Bulk #C1-C3 are different between **(a)** and **(b)**.

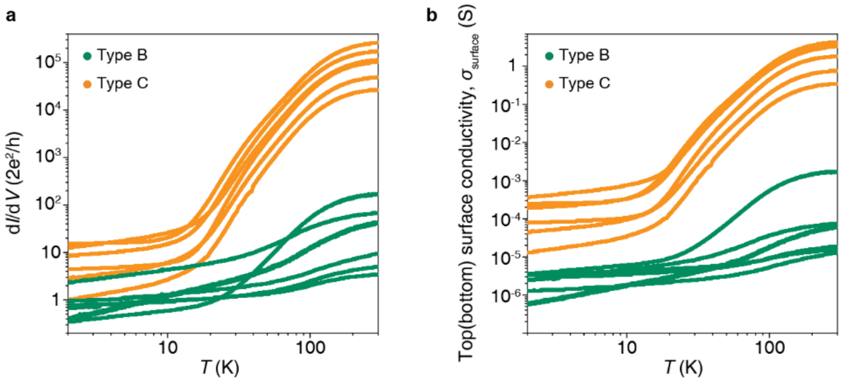

Supplementary Fig. 10 Temperature dependence d*I*/d*V* (a) and top(bottom) surface conductivity $\sigma_{surface}$ (b, defined as conductance / width × length) for type B (green line) and C (orange line) samples. The curves in **(b)** diverge a lot in Luttinger liquid behavior region, which can exclude that Luttinger liquid behavior arises from top and/or bottom surface of $Ta_2Pd_3Te_5$ thin film devices/bulk samples.

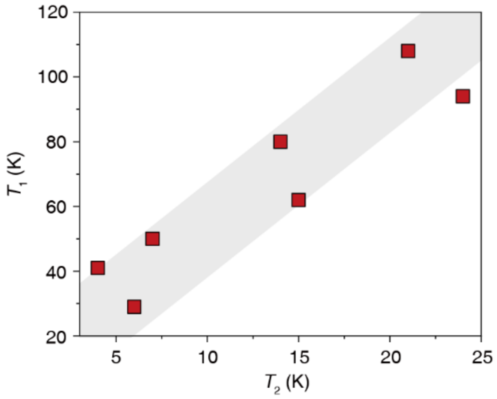

Supplementary Fig. 11 T_1 versus T_2 for type A samples. It shows approximate linear behavior. Uncertainties in both *x* and *y*-axis are smaller than point size.

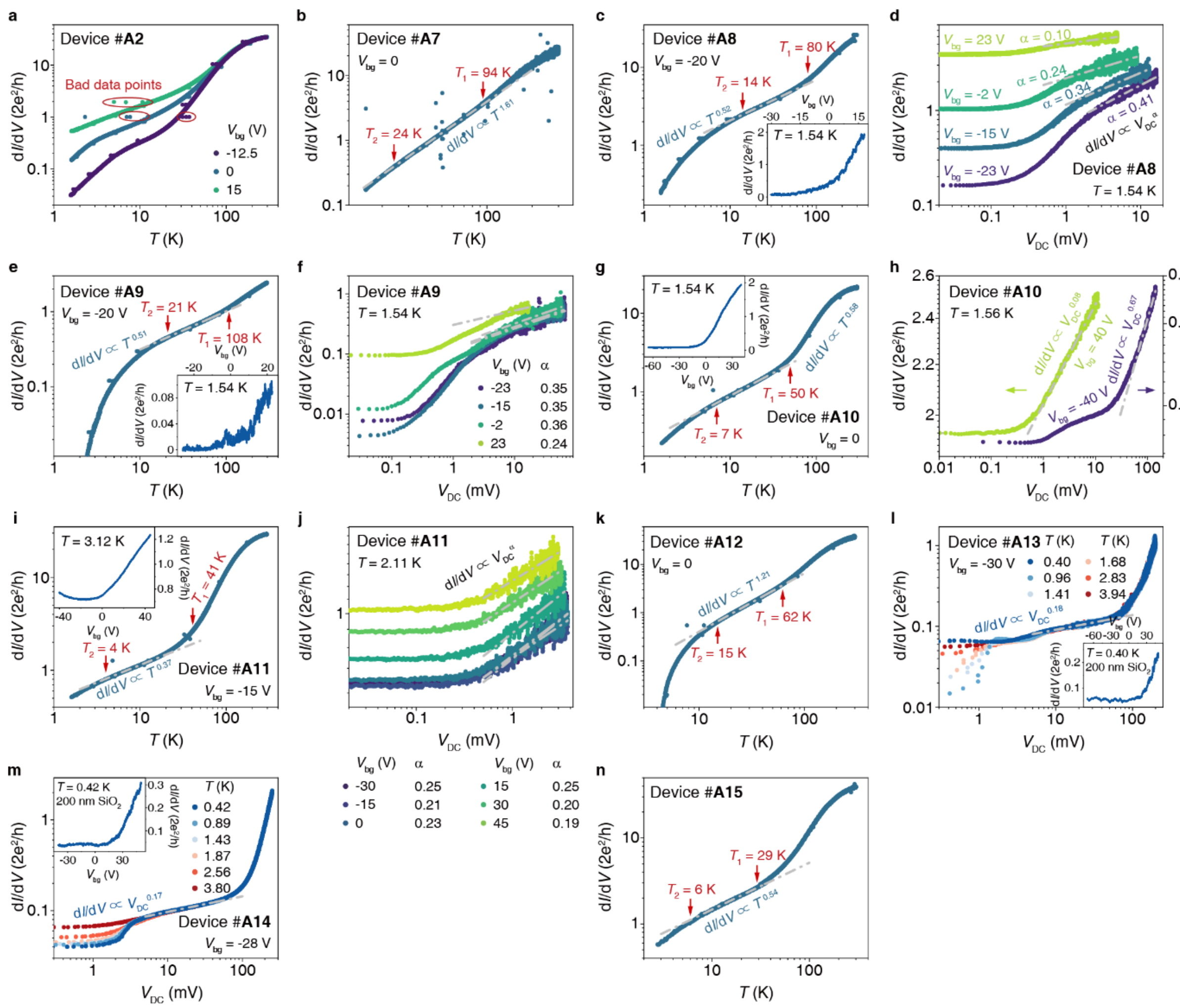

Supplementary Fig. 12 Original data for type A devices. a,b, Original temperature dependence dI/dV data of device #A2 **(a)** and device #A7 **(b)**. (Bad data points due to thermometer range switches are removed in dI/dV (T) curves in main figures and raw data are shown in Supplementary Figures. These bad data points don't affect the trend of dI/dV (T) curves.) **c,d,** dI/dV versus T **(c)** and V_{DC} **(d)** of device #A8 with the inset shows gate tunable dI/dV at T = 1.54 K. **e,f,** dI/dV versus T **(e)** and V_{DC} **(f)** of device #A9 with the inset shows gate tunable dI/dV at T = 1.54 K. **g,h,** dI/dV versus T **(g)** and V_{DC} **(h)** of device #A10 with the inset shows gate tunable dI/dV at T = 1.54 K. **i,j,** dI/dV versus T **(i)** and V_{DC} **(j)** of device #A11 with the inset shows gate tunable dI/dV at T = 3.12 K. **k,** Temperature dependence dI/dV of device #A12. **l,** dI/dV versus V_{DC} of device #A13 with the inset shows gate tunable dI/dV at T = 0.40 K. **m,** dI/dV versus V_{DC} of device #A14 with the inset shows gate tunable dI/dV at T = 0.42 K. **n,** Temperature dependence dI/dV of device #A15. The gray dot dash lines show power-law behaviors.

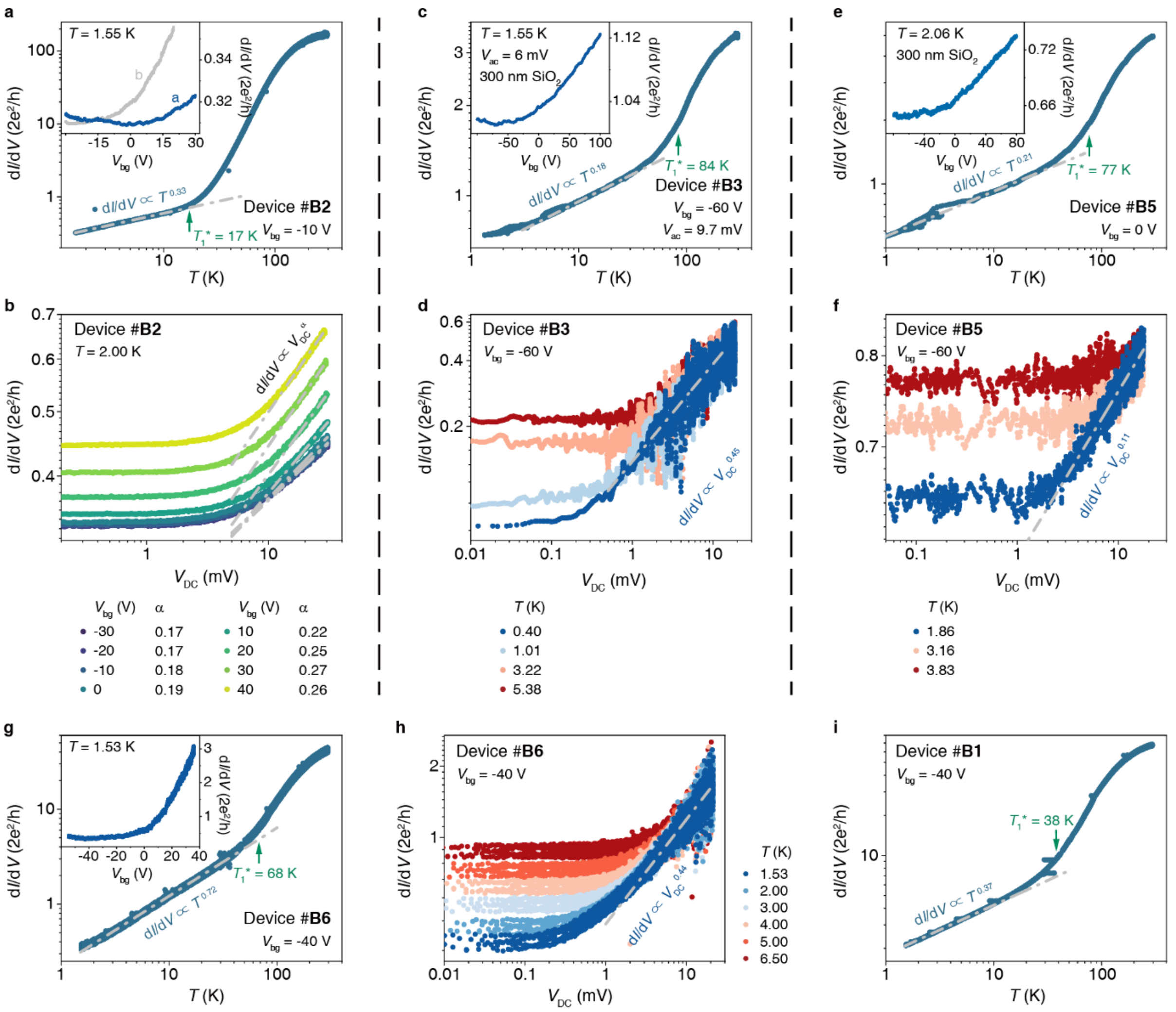

Supplementary Fig. 13 Original data for type B devices. a,b, dI/dV versus T **(a)** and V_{DC} **(b)** of device #B2 with the inset shows gate tunable dI/dV at T = 1.55 K. Note that the CNP changed after several times of cooldown, then the blue line 'a' in the inset is for **(a)** and gray line 'b' for **(b)**. **c,d,** dI/dV versus T **(c)** and V_{DC} **(d)** of device #B3 with the inset shows gate tunable dI/dV at T = 1.55 K. Note that the slight saturation in the lowest temperature region in dI/dV versus T is due to large a.c. voltage we applied. **e,f,** dI/dV versus T **(e)** and V_{DC} **(f)** of device #B5 with the inset shows gate tunable dI/dV at T = 2.06 K. **g,h,** dI/dV versus T **(g)** and V_{DC} **(h)** of device #B6 with the inset shows gate tunable dI/dV at T = 1.53 K. **i,** Original temperature dependence dI/dV data of device #B1.

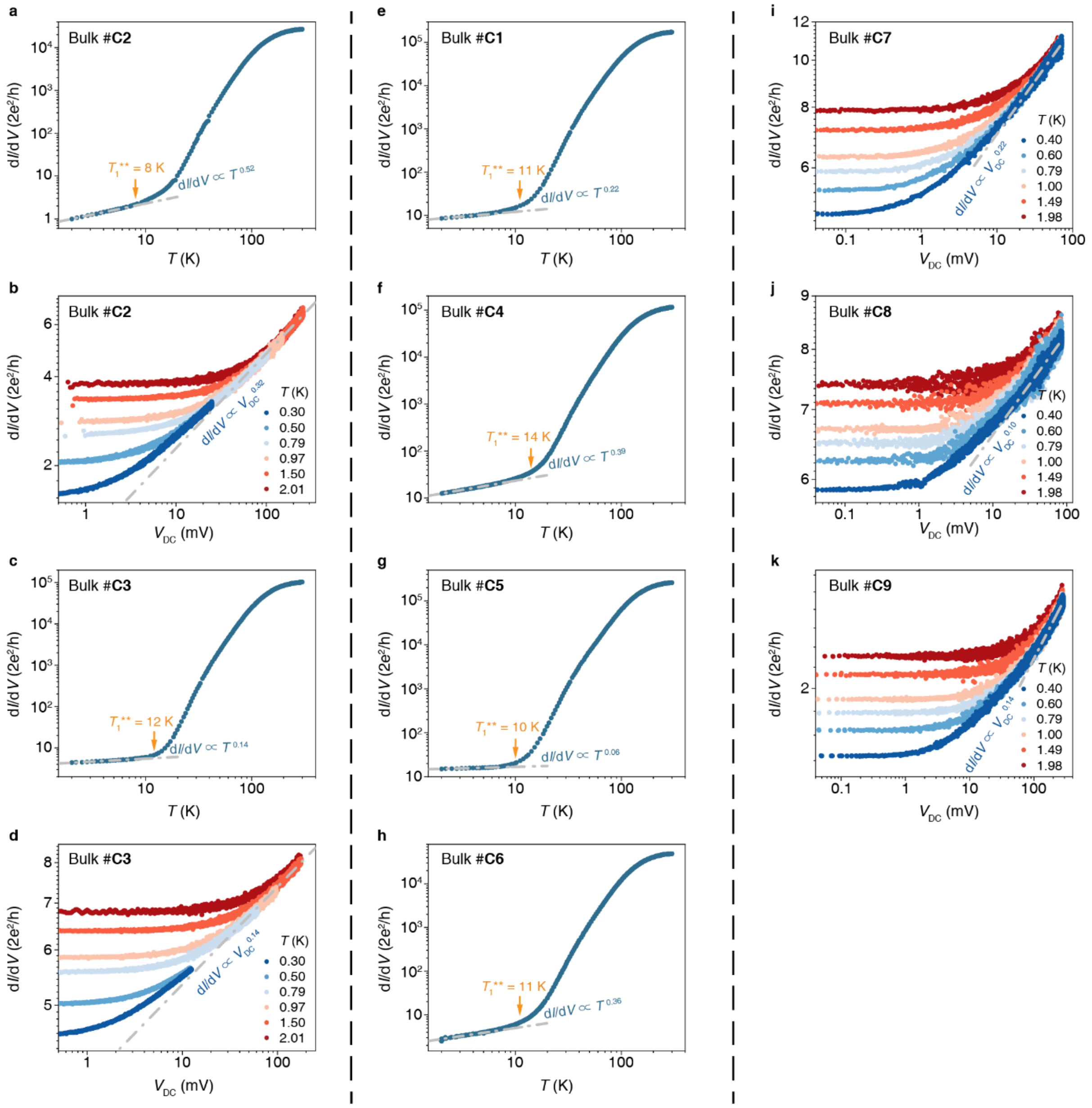

Supplementary Fig. 14 Original data for type C bulk samples. a,b, dI/dV versus T **(a)** and V_{DC} **(b)** of bulk sample #C2. **c,d,** dI/dV versus T **(c)** and V_{DC} **(d)** of bulk sample #C3. **e-h,** Temperature dependence dI/dV of bulk samples #C1 **(e)**, #C4 **(f)**, #C5 **(g)**, and #C6 **(h)**. Note that due to different leads spacing in two measurements, the values of dI/dV are slightly different between **(e)** and Fig. 5f. **i-k,** dI/dV versus V_{DC} of bulk samples #C7 **(i)**, #C8 **(j)**, and #C9 **(k)**.
